# Supplementary material for: Vitamins D3 and D2 have marked but different global effects on gene expression in a rat oligodendrocyte precursor cell line
Source: Mol Med. 2020 Apr 9;26:32. doi: 10.1186/s10020-020-00153-7 (PMC7146914; doi:10.1186/s10020-020-00153-7)
Supplement: Supplementary file 3 — Additional file 3: Supplementary Fig. S1. Functional enrichment analysis of the genes down-regulated by vitamin D. Supplementary Fig. S2. Functional enrichment analysis of the genes up-regulated by vitamin D (PDF 935 KB). [file 10020_2020_153_MOESM3_ESM.pdf]

Additional File 3: Supplementary Figures S1 and S2

This PDF file includes:

Supplementary Figure S1. Functional enrichment analysis of the genes down-regulated by vitamin D.

Supplementary Figure S2. Functional enrichment analysis of the genes up-regulated by vitamin D.

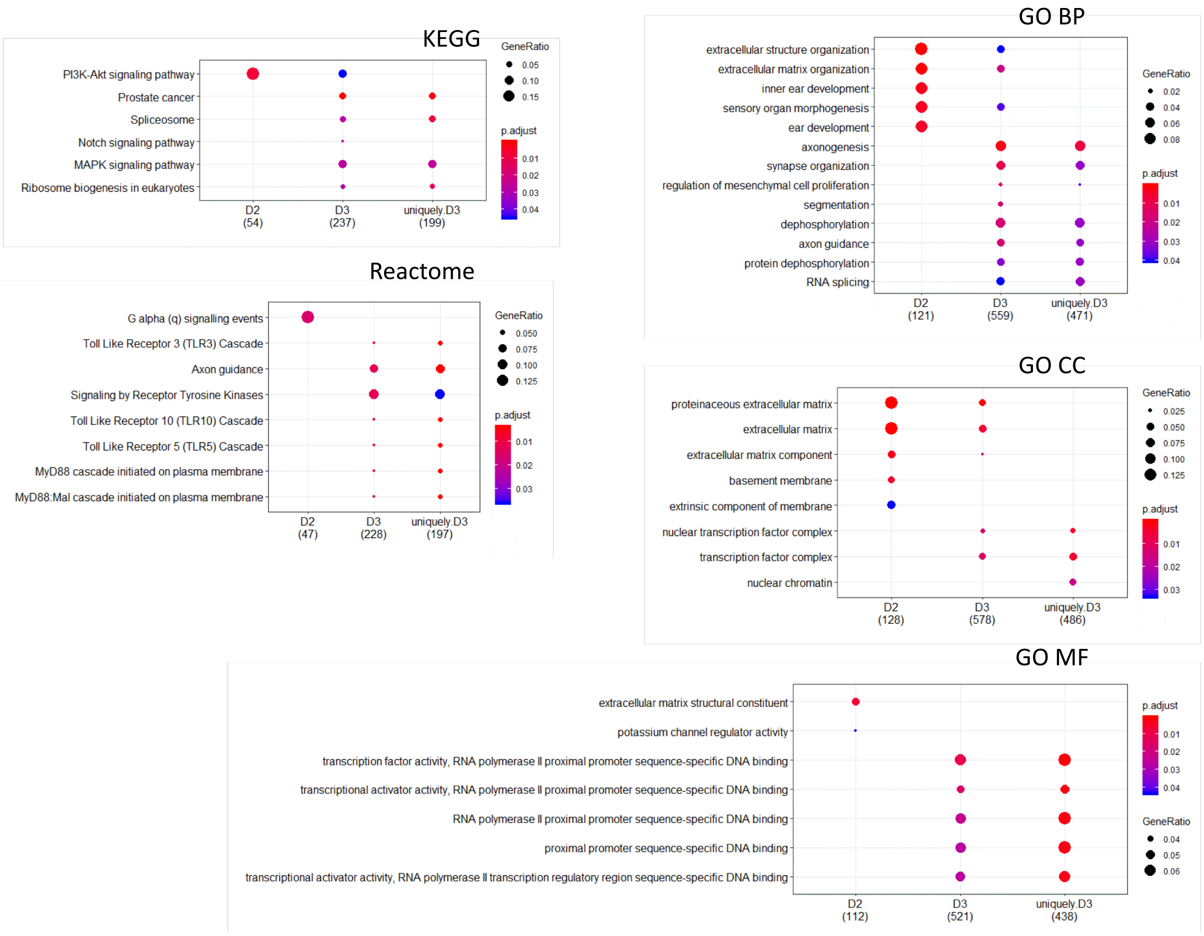

Supplementary Figure S1. **Functional enrichment analysis of the genes down-regulated by vitamin D.** Comparative functional enrichment analysis of the genes corresponding to the probes significantly decreasing in abundance after 24 h in response to either vitamin D2 (D2) or D3 (D3) and of those uniquely downregulated by D3 (uniquely.D3). Only the top significant categories are shown. Full results are provided in Supplementary File S3.

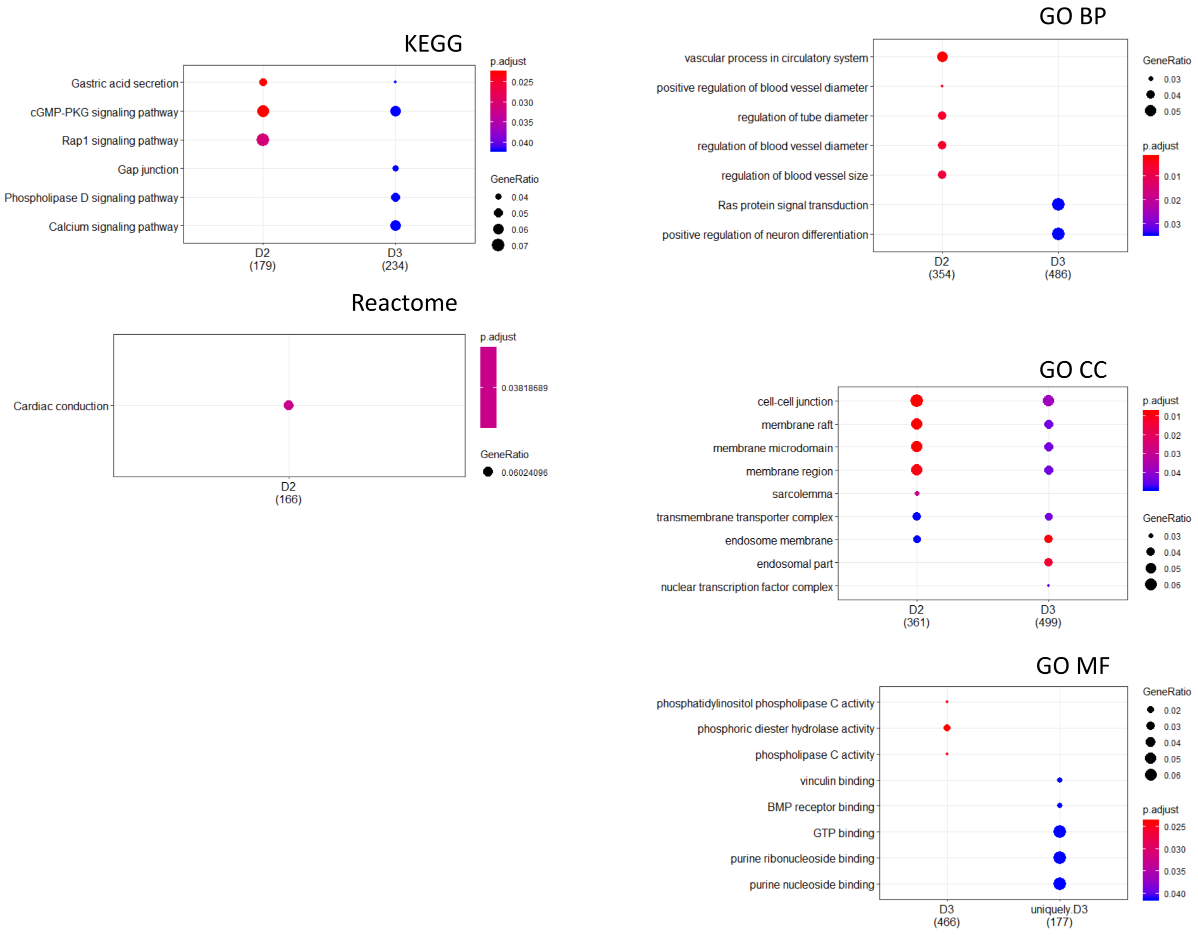

Supplementary Figure S2. **Functional enrichment analysis of the genes up-regulated by vitamin D.** Comparative functional enrichment analysis of the genes corresponding to the probes significantly increasing in abundance after 24 h in response to either vitamin D2 (D2) or D3 (D3) and of those uniquely downregulated by D3 (uniquely.D3). Only the top significant categories are shown. Full results are provided in Supplementary File S3.
