# Supplementary material for: Vitamins D3 and D2 have marked but different global effects on gene expression in a rat oligodendrocyte precursor cell line
Source: Mol Med. 2020 Apr 9;26:32. doi: 10.1186/s10020-020-00153-7 (PMC7146914; doi:10.1186/s10020-020-00153-7)
Supplement: Supplementary file 5 — Additional file 5: Supplementary Table S1. RT-qPCR validation of microarray data (PDF 29 KB). [file 10020_2020_153_MOESM5_ESM.pdf]

Additional File 5: Supplementary Table S1. RT-qPCR validation of microarray data.

	Microarrays				qPCR			
Gene	D2 vs Ctrl		D3 vs Ctrl		D2 vs Ctrl		D3 vs Ctrl	
	Log_2 FC	Adj.P.Val	Log_2 FC	Adj.P.Val	Log_2 FC	*P* value	Log_2 FC	*P* value
Jag1	-0.39	0.02701	-0.50	7.1E-04	-0.42	0.00021	-0.56	0.00074
Dll3	-0.49	0.00055	-0.41	0.00539	-0.27	0.04417	-0.22	0.02117
Psen1	ns	-	-0.61	0.00068	ns	-	ns	-
Rras2	0.90	2.2E-12	0.79	5.6E-11	1.89	0.00057	1.90	0.00536
Ngf	0.55	0.00061	0.98	1.1E-08	2.01	8.5E-06	2.49	1.18E-06
Tgfb2	ns	-	0.46	0.02564	ns	-	0.61	0.03340
Sox4	ns	-	-0.38	0.01949	ns	-	-0.24	0.01877
Tfeb	ns	-	-0.38	0.01989	-0.16	0.03248	-0.37	0.00144
VDR	0.78	5.5E-06	0.69	0.00004	1.92	3.5E-06	1.88	1.2E-06

Results for nine genes are shown, comparing expression data from microarrays with respective results from qPCR at 24 h after treatment with vitamin D2 or D3. Results are expressed as log base 2 ratio vs control (Ctrl) and are the mean of quadruplicate samples. Adjusted p-values (Adj.p.val) for microarray data analysis were obtained using LIMMA and *P* values for qPCR data analysis were calculated by Student’s t-test.
